# Differences in Number of Midbrain Dopamine Neurons Associated with Summer and Winter Photoperiods in Humans

**DOI:** 10.1371/journal.pone.0158847

**Published:** 2016-07-18

**Authors:** Tim D. Aumann, Mai Raabus, Doris Tomas, Agustinus Prijanto, Leonid Churilov, Nicholas C. Spitzer, Malcolm K. Horne

**Affiliations:** 1 Florey Institute of Neuroscience and Mental Health, The University of Melbourne, Parkville, Victoria, 3010, Australia; 2 Neurobiology Section, Division of Biological Sciences and Center for Neural Circuits and Behavior, University of California San Diego, La Jolla, California, 92093–0357, United States of America; 3 Kavli Institute for Brain and Mind, University of California San Diego, La Jolla, California, 92093–0357, United States of America; Kent State University, UNITED STATES

## Abstract

Recent evidence indicates the number of dopaminergic neurons in the adult rodent hypothalamus and midbrain is regulated by environmental cues, including photoperiod, and that this occurs via up- or down-regulation of expression of genes and proteins that are important for dopamine (DA) synthesis in extant neurons (‘DA neurotransmitter switching’). If the same occurs in humans, it may have implications for neurological symptoms associated with DA imbalances. Here we tested whether there are differences in the number of tyrosine hydroxylase (TH, the rate-limiting enzyme in DA synthesis) and DA transporter (DAT) immunoreactive neurons in the midbrain of people who died in summer (long-day photoperiod, n = 5) versus winter (short-day photoperiod, n = 5). TH and DAT immunoreactivity in neurons and their processes was qualitatively higher in summer compared with winter. The density of TH immunopositive (TH+) neurons was significantly (~6-fold) higher whereas the density of TH immunonegative (TH-) neurons was significantly (~2.5-fold) lower in summer compared with winter. The density of total neurons (TH+ and TH- combined) was not different. The density of DAT+ neurons was ~2-fold higher whereas the density of DAT- neurons was ~2-fold lower in summer compared with winter, although these differences were not statistically significant. In contrast, midbrain nuclear volume, the density of supposed glia (small TH- cells), and the amount of TUNEL staining were the same in summer compared with winter. This study provides the first evidence of an association between environmental stimuli (photoperiod) and the number of midbrain DA neurons in humans, and suggests DA neurotransmitter switching underlies this association.

## Introduction

Changes in the brain or ‘brain plasticity’ underlie adaptive behavior and brain repair following injury or disease. Recently, a new form of brain plasticity was identified in which neurons appear to switch on and off expression of proteins important for dopamine (DA) synthesis and neurotransmission in response to environmental cues; a phenomenon we term ‘DA neurotransmitter switching’. Specifically, adult rats exposed to less stressful short-day photoperiods have more hypothalamic dopamine (DA) neurons, whereas those exposed to more stressful long-day photoperiods have fewer hypothalamic DA neurons [1]. These changes are not associated with neurogenesis or apoptosis. Moreover, ablating hypothalamic DA neurons results in anxious and depressed behavior, and this is rescued specifically by photoperiod-induction of more DA neurons [1].

Sustained exposure to bright light also reduces the number of tyrosine hydroxylase (TH, the rate limiting enzyme in DA synthesis) immunopositive (TH+) neurons in the substantia nigra pars compacta (SNc), as well as DA levels in the striatum (a target of SNc DA neurons), in adult rats [2], potentially reflecting a difference between bright artificial and natural levels of illumination. Other environmental stimuli also change the number of SNc (and ventral tegmental area) TH+ neurons. Specifically, pairing male and female mice together continuously for 7 days results in more TH+ neurons in males (compared with male-male pairs) but fewer TH+ neurons in females (compared with female-female pairs) [3,4]. Alternatively, housing male mice in enriched environments (EE) for 14 days results in more TH+ neurons (compared with standard-housed mice) [3,4]. Furthermore, EE-induction of more midbrain TH+ neurons is abolished by concurrent local infusion of GABA_A_ receptor antagonists, implicating afferent pathways, synaptic input and neuronal activity in mediating this effect [3,4].

Indeed targeting the activity of midbrain neurons by administering ion-channel agonists or antagonists to adult mice for 2 weeks increases or decreases (depending on the drug) the number of SNc TH+ neurons without changing the total number (TH+ and TH- combined) of SNc neurons [5,6]. This indicates DA neurotransmitter switching, because adult midbrain neurogenesis and DA neurogenesis occur either not at all or at rates too low to account for the magnitude and speed of these changes [7–14].

Environment may regulate DA levels and DA signaling in the adult human brain also. Seasonal variations in the concentration of DA, DA metabolites, DA receptors and the DA transporter (DAT) have been detected in cerebrospinal fluid [15,16], hypothalamus [17,18] and striatum [19–22] in living and deceased subjects. As low DA levels contribute to depression (reviewed [23]), seasonal affective disorder (SAD) or the ‘winter blues’ may be due to low levels of DA caused by sustained decreases in light stimulation. Indeed, striatal DAT availability appears reduced in un-medicated depressed SAD patients [24], bright light therapy improves mood in SAD patients (reviewed in [25]), and inhibiting DA synthesis in SAD patients during summer remission restores depressive symptoms [26,27].

Some diurnal rodents react to short days as humans do, with increased depression-like behavior [28], while some nocturnal rodents (C57BL/6J and Siberian hamsters) react to short days as humans do, with increased depression-like behavior. The basis for the similarity between these nocturnal and diurnal rodent responses and human responses in some cases but not in others is currently unknown. Diurnal and nocturnal rodents are put in a single category largely on the basis of the time of day when they are most active, but the nocturnality of one set of rodents may not be the same as the nocturnality of another set. Differences in circuit wiring could provide the explanation, and future circuit tracing experiments (e.g. with rabies virus; [29]) can address this issue.

Similarities between these rodent and human findings led us to ask whether environment regulates the number of DA neurons in the human midbrain. If so, one might expect different numbers of midbrain DA neurons in summer (long photoperiod) compared with winter (short photoperiod). To test this we examined midbrain TH and DAT immunoreactivities in people who lived at high latitude (Scotland) but died in summer or winter. Here we report the first evidence of an association between environmental stimuli (photoperiod) and the number of midbrain DA neurons in humans. Our findings also suggest DA neurotransmitter switching underlies this association.

## Materials and Methods

### Subjects

All analyses were carried out on human post-mortem brain tissue that had been fixed in formaldehyde for 3–5 days before being processed to paraffin wax blocks. The blocks were supplied by the Medical Research Council (MRC)-funded Edinburgh University Brain Bank. The study was approved by the MRC-funded Edinburgh University Brain Bank Review Board, with the verbal and written consent of the families of the deceased; and was conducted according to the principles expressed in the Declaration of Helsinki.

All subjects were living in Scotland at latitudes of ~55° North at the time of death, which was either summer (n = 5, June 1^st^ to August 31^st^) or winter (n = 5, November 1^st^ to January 31^st^). These dates correspond to daylight exposure of approximately 17 hr 13 min (June 1^st^) to 13 hr 54 min (August 31^st^), with a maximum of 17 hr 36 min (June 21^st^), in the summer group; and 9 hr 12 min (November 1^st^) to 8 hr 34 min (January 31^st^), with a minimum of 6 hr 57 min (December 22^nd^), in the winter group. Other subject information is in Table 1.

**Table 1:** Summer (top) and winter (bottom) subject information.

Subject^a^	Date of death^c^	Time of death^d^	Age	Gender	PMI^e^	Cause of death
SD030/11	07/23/2011	12:12	30	M	71	Unascertained
SD032/11	08/10/2011	16:28	58	M	43	Acute myocardial infarction, coronary artery thrombosis, coronary artery atherosclerosis
SD033/11	08/24/2011	05:20	72	F	30	Hemopericardium, ruptured myocardial infarct, coronary artery thrombosis, coronary artery atherosclerosis
SD030/12	06/24/2012	18:20	71	F	41	Ischemic and hypertensive heart disease
SD037/12	08/06/2012	16:55	52	M	91	Chest injuries, road traffic collision
Summer^b^ mean (SE)	272 (10)	831 (142)	56.6 (7.7)		55.2 (11.2)	
SD002/10	01/17/2010	10:24	38	M	49	Right ventricular hypertrophy
SD003/10	01/21/2010	01:45	65	M	34	Diabetic keto-acidosis and morphine toxicity, Ischemic heart disease
SD034/10	11/01/2010	10:55	49	F	49	Metastatic carcinoma of breast
SD001/11	01/16/2011	13:16	74	M	46	Pulmonary thrombo-embolism, deep venous thrombosis
SD002/12	01/30/2012	22:04	67	M	61	Ruptured atherosclerotic abdominal aneurysm, hypertensive and ischemic heart disease
Winter^b^ mean (SE)	66 (16)*	701 (195)^ns^	58.6 (6.6)^ns^		47.8 (4.3)^ns^	

^a^All subjects were Scottish nationals residing in Scotland at the time of death.

^b^Summer subjects died between June 1^st^ and August 31^st^; winter subjects died between November 1^st^ to January 31^st^. These dates correspond to daylight exposure of approximately 17 hr 13 min (June 1^st^) to 13 hr 54 min (August 31^st^), with a maximum of 17 hr 36 min (June 21^st^), in the summer group; and 9 hr 12 min (November 1^st^) to 8 hr 34 min (January 31^st^), with a minimum of 6 hr 57 min (December 22^nd^), in the winter group.

^c^Date of death is listed as mm/dd/yyyy for each subject, but was converted to number of days after 11/01 of each year for the statistical comparison and mean (SE) of the summer and winter groups.

^d^Time of death is listed as hh:mm for each subject, but was converted to number of minutes after 00:00 of each day for the statistical comparison and mean (SE) of the summer and winter groups.

^e^PMI = post-mortem interval (hr) before brains were formaldehyde-preserved

* = significant (p<0.05) difference between summer and winter

^ns^ = not significant (p>0.05) between summer and winter; unpaired, two-tailed t-tests.

### TH and DAT immunohistochemistry for stereology

Serial sections (8 μm thick) were cut from each block, mounted on microscope slides, and stored in the dark at 21°C. A series of sections (1:50; i.e. 400 μm apart) through the entire midbrain block of each and every subject were then processed at the same time for TH or DAT immunohistochemistry as follows. Paraffin wax was removed by placing sections in an oven at 62°C for 30 min, followed by 100% xylene (3 x 5 min). Sections were next hydrated through decreasing concentrations of ethanol [1 min each in 100%, 100%, 90%, 70%, 50%, and 0% (tap water)]. Endogenous peroxidase activity was quenched by placing sections in 5% H_2_O_2_ in distilled water for 5 min. Sections were washed [(3 x 5 min in phosphate buffered saline (PBS)] and antigen retrieval was performed in citrate buffer (0.2%, pH 6.0, 98°C, 2 hr). Sections were placed in primary antibody solution comprising 1:1000 rabbit anti-TH (Cat# 657012, Millipore, Temecula, CA, USA) or 1:500 rat anti-DAT (Cat# MAB369, Millipore, Temecula, CA, USA), 1% normal goat (for TH) or rabbit (for DAT) serum, 0.3% Triton X-100, and PBS at 4°C for 48 hr. Both these primary antibodies have specificity in human brain tissue. Sections were washed at 21°C (2 x 5 min in PBS) then placed in blocking solution comprising 6% normal goat (for TH) or rabbit (for DAT) serum, 0.3% Triton X-100, and PBS for 15 min. Sections were next placed in secondary antibody solution comprising 1:1000 goat anti-rabbit IgG conjugated to avidin-peroxidase (DAKO, Glostrup, Denmark) for TH or 1:500 rabbit anti-rat IgG conjugated to avidin-peroxidase (Vector, Burlingame, CA, USA) for DAT, 1% goat serum (for TH) or 1% rabbit serum (for DAT), 0.3% Triton X-100, and PBS at 21°C for 2 hr. Sections were washed (3 x 5 min in PBS) and placed in diamino-benzidine (DAB) solution comprising 1% DAB, 1% cobalt chloride and 1% nickel sulphate in PBS (filtered before use) at 21°C for 20 min. Three percent H_2_O_2_ was added to the DAB solution on the sections for another 2 min before three further washes (5 min each) in PBS. Once dry, sections were counterstained for Nissl substance (1% neutral red, 10 min) and dehydrated (5 sec each) through increasing concentrations of alcohol [0% (tap water), 50%, 70%, 90%, 100%, 100%], cleared in X3B (Oilchem, Epping, Victoria, Australia) and coverslipped.

### TH immunohistochemistry and TUNEL staining

Sections were washed and antigen retrieval performed as detailed above. Next, tissue auto-fluorescence was suppressed by incubating sections in 70% ethanol (5 min), auto-fluorescence eliminator (Millipore, Billerica, MA, USA; 5 min), 70% ethanol (3 x 1 min) then PBS (5 min). Antibody incubations were performed as above (for TH) except the secondary antibody used was donkey anti-rabbit 488 (1:100; Jackson ImmunoResearch, West Grove, PA, USA) and normal donkey serum was used instead of normal goat serum. Following secondary antibody incubation, sections were washed in PBS (3 x 5 min).

TUNEL staining was then performed using an In Situ Cell Death Detection Kit (Roche, Castle Hill, NSW, Australia). Sections were further incubated in the prescribed mixture of enzyme and label solution at 37°C for 60 min in a humidified chamber. Positive control was provided by prior incubation at 21°C for 10 min with DNase I recombinant solution [1500 U/ml DNase I (Roche, Castle Hill, NSW, Australia) in 10 mM Tris-HCl pH 7.5, 1 mM MgCl_2_ and 1 mg/ml bovine serum albumin] to induce DNA breaks. Negative control was obtained by incubating in the label solution without the enzyme component. Sections were washed in PBS (3 x 5 min) and coverslipped in fluorescence mounting medium while wet (Dako, North Sydney, NSW, Australia).

### Stereology

To measure the density of TH+, TH-, DAT+ and DAT- midbrain neurons we used a Leica DMLB microscope fitted with a motorized X-Y stage and Stereo Investigator software (ver 11.03.1 64bit, MicroBrightField Inc., Williston VT, USA). Each immuno-processed section was examined and, if present, midbrain DA nuclei were identified and outlined at low magnification (x80) according to the presence of TH+ and neuromelanin+ (or DAT+ and neuromelanin+) neurons. Note the A8 (retrorubral field), A9 (SNc) and A10 (ventral tegmental area) midbrain DA nuclear groups were treated as a single entity in these analyses. A counting frame measuring 400 μm x 400 μm was placed randomly and systematically at intervals of x = 933.6 μm and y = 803.1 μm throughout these outlined nuclei. Thus, the sampling fraction of each section was (400 x 400)/(933.6 x 803.1) = 21.3%, and the sampling fraction of each subject was 21.3/50 = 0.43% (i.e. every 50^th^ section). At each counting frame location (counting site; n = 42–207 per subject, Tables 2 & 3), every neuron (large cell with a visible nucleus) in the counting frame was scored as TH+ (or DAT+) (presence of black DAB immunohistochemistry reaction product in the cytoplasm) or TH- (or DAT-) (no immunohistochemistry reaction product but neuromelanin+ and/or red Nissl stain) at high magnification (x320). The same analyst performed these measurements on all subjects and was blind to group identity (summer or winter). Cell density for each subject was calculated as the average cell density across all counting sites for each subject.

**Table 2:** TH+ and TH- neuron densities in summer (top) and winter (bottom) subjects.

Subject	Block location^a^	Block size (WxHxD)^b^	Sections analyzed	Counting sites analyzed	TH+ neuron density (/mm^2^)	TH- neuron density (/mm^2^)	Total neuron density (/mm^2^)
SD030/11	3	28x21x1.9	4	88	41.7	7.1	48.8
SD032/11	3	31x23x2.5	4	126	22.8	13.2	36.0
SD033/11	2	32x21x3.1	5	160	35.2	9.0	44.2
SD030/12	2	28x21x3.6	6	183	34.1	9.2	43.3
SD037/12	4	21x24x2.2	4	53^c^	34.7	7.3	42.0
Summer mean (SE)	2.8 (0.4)	28x22x2.7	4.6 (0.4)	122.0 (23.6)	33.7 (3.1)	9.2 (1.1)	42.9 (2.1)
SD002/10	2	30x24x2.2	6	199	7.3	30.1	37.4
SD003/10	2	32x22x2.2	4	133	0.6	35.9	36.5
SD034/10	4	27x22x2.7	5	174	0.2	12.9	13.1
SD001/11	4	27x22x1.2	3	57^c^	4.4	15.4	19.8
SD002/12	3	16x25x2.1	6	151	15.2	21.9	37.1
Winter mean (SE)	3.0 (0.4)^d^	26x23x2.1	4.8 (0.6)^e^	142.8 (24.1)^e^	5.5 (2.7)^f^	23.2 (4.3)^f^	28.7 (5.2)^g^

^a^Estimated as: 1 = rostral-most quarter, 2–4 = next three quarters moving progressively caudally.

^b^Block size (mm) measured at maximum width (W = medio-lateral) and height (H = dorso-ventral). D = depth = rostro-caudal.

^c^Number of counting sites is low due to a low volume of midbrain DA nuclei within the block provided.

^d^Rostro-caudal block location is not significantly different in summer compared with winter (p>0.05, unpaired, two-tailed t-test).

^e^The number of sections and number of counting sites are not significantly different in summer compared with winter (p>0.05, unpaired, two-tailed t-tests).

^f^TH+ neuron density is ~6-fold higher (on average) and TH- neuron density is ~2.5-fold lower in summer compared with winter. These differences are significantly different (p<0.017, unpaired, two-tailed t-tests with correction for multiple comparisons).

^g^Total neuron density (TH+ & TH- combined) is not significantly different (p>0.017, unpaired two-tailed t-test with correction for multiple comparisons) in summer compared with winter.

**Table 3:** DAT+ and DAT- neuron, and glial cell densities in summer (top) and winter (bottom) subjects.

Subject	Sections analyzed	Counting sites analyzed	DAT+ neuron density (/mm^2^)	DAT- neuron density (/mm^2^)	Total neuron density (/mm^2^)	glial cell density (/mm^2^)^e^
SD030/11	4	119	30.4	1.3	31.7	881.3
SD032/11	4	110	28.9	1.5	30.4	759.0
SD033/11	4	133	16.5	9.1	25.6	1023.6
SD030/12	6	173	37.5	7.0	44.5	943.8
SD037/12	4	42^a^	37.4	10.1	47.5	1020.8
Summer mean (SE)	4.4 (0.4)	115.4 (21.3)	30.1 (3.8)	5.8 (1.9)	35.9 (4.3)	925.7 (49.4)
SD002/10	5	207	29.4	4.4	33.8	780.6
SD003/10	4	206	1.6	22.1	23.7	738.2
SD034/10	5	80	2.1	2.3	4.4	872.2
SD001/11	3	45^a^	16.5	1.8	18.3	1340.3
SD002/12	4	97	28.4	21.3	49.7	747.9
Winter mean (SE)	4.2 (0.4)^b^	127.0 (33.5)^b^	15.6 (6.1)^c^	10.4 (4.7)^c^	26.0 (7.6)^d^	895.8 (113.6)^e^

^a^Number of counting sites is low due to a low volume of midbrain DA nuclei within the block provided.

^b^The number of sections and number of counting sites are not significantly different in summer compared with winter (p>0.05, unpaired, two-tailed t-tests).

^c^Although DAT+ neuron density is ~2-fold higher (on average) and DAT- neuron density is ~2-fold lower in summer compared with winter, these differences are not significantly different (p>0.017, unpaired, two-tailed t-tests with correction for multiple comparisons).

^d^Total neuron density (DAT+ & DAT- combined) is not significantly different (p>0.017, unpaired two-tailed t-test with correction for multiple comparisons) between summer and winter.

^e^Glial cell density is not significantly different in summer compared with winter (p>0.05, unpaired, two-tailed t-tests). Note, the sections analyzed and counting sites analyzed columns are not applicable to the glial cell density column in this table. Glial cell density was measured in a separate analysis using different stereological sampling parameters as detailed in the Materials and Methods section.

We also measured the density of presumed glial cells, which were identified as small Nissl+ cells with a visible nucleus that were also TH- and neuromelanin-. They were measured in the same sections analyzed for TH. However, due to their smaller size, higher density, and the need to analyze them at higher power magnification (x640), the counting frame was reduced in size to 120 μm x 120 μm and it was placed randomly and systematically at intervals of x = 500 μm and y = 500 μm, resulting in a sampling fraction/section of (120 x 120)/(500 x 500) = 5.8%, and a sampling fraction/subject of 5.8/50 = 0.12%.

### Statistics

Unpaired, two-tailed t-tests were used to compare the means of measures from subjects across different groups (summer versus winter). The significance level was set at p = 0.05, and corrections were made in situations where multiple t-tests were applied to dependent measures.

In addition, due to the non-independent nature of some of the measures on the same subject (e.g. TH+ neurons, TH- neurons and total neurons), to estimate the difference between the seasons of death, we have used clustered median regression with individual subjects treated as a cluster. Season of death (summer or winter) was treated as an independent variable, the corresponding cell density in a given area (e.g. section and side of the brain) as a dependent variable, and subject age, gender, side of the brain and PMI as adjustment covariates.

## Results

### Subject comparisons

The subject information detailed in Table 1 shows there was a significant difference in the date of death, but no significant differences in time of death, age, gender or post-mortem interval (PMI) between the summer and winter groups. Cause of death was cardiovascular and non-neurological in most cases (Table 1).

### Midbrain comparisons

The supplied tissue blocks comprised the entire dorso-ventral and medio-lateral extent of the midbrain bilaterally, except for one subject (SD002/12) in which only one side of the midbrain was supplied. In contrast, the blocks comprised only a fraction of the rostro-caudal extent of the midbrain DA nuclei. Specifically, our blocks comprised 1.2–3.6 mm (2.4 mm on average) of the midbrain rostro-caudally (Table 2), whereas the entire midbrain DA nuclei extend ~10 mm rostro-caudally [30]. Thus, approximately 12–36% (24% on average) of the entire midbrain DA nuclei was sampled from each subject. Importantly, there were no differences in the rostro-caudal locations of the blocks, the size of the blocks, the number of sections analyzed, and the number of counting sites analyzed between the summer and winter groups (Tables 2 & 3), indicating that the regions of midbrain DA nuclei, midbrain volume, and midbrain DA nuclear volume were the same in the summer and winter groups.

### Qualitative TH and DAT immunoreactivity comparisons

**Fig 1:**
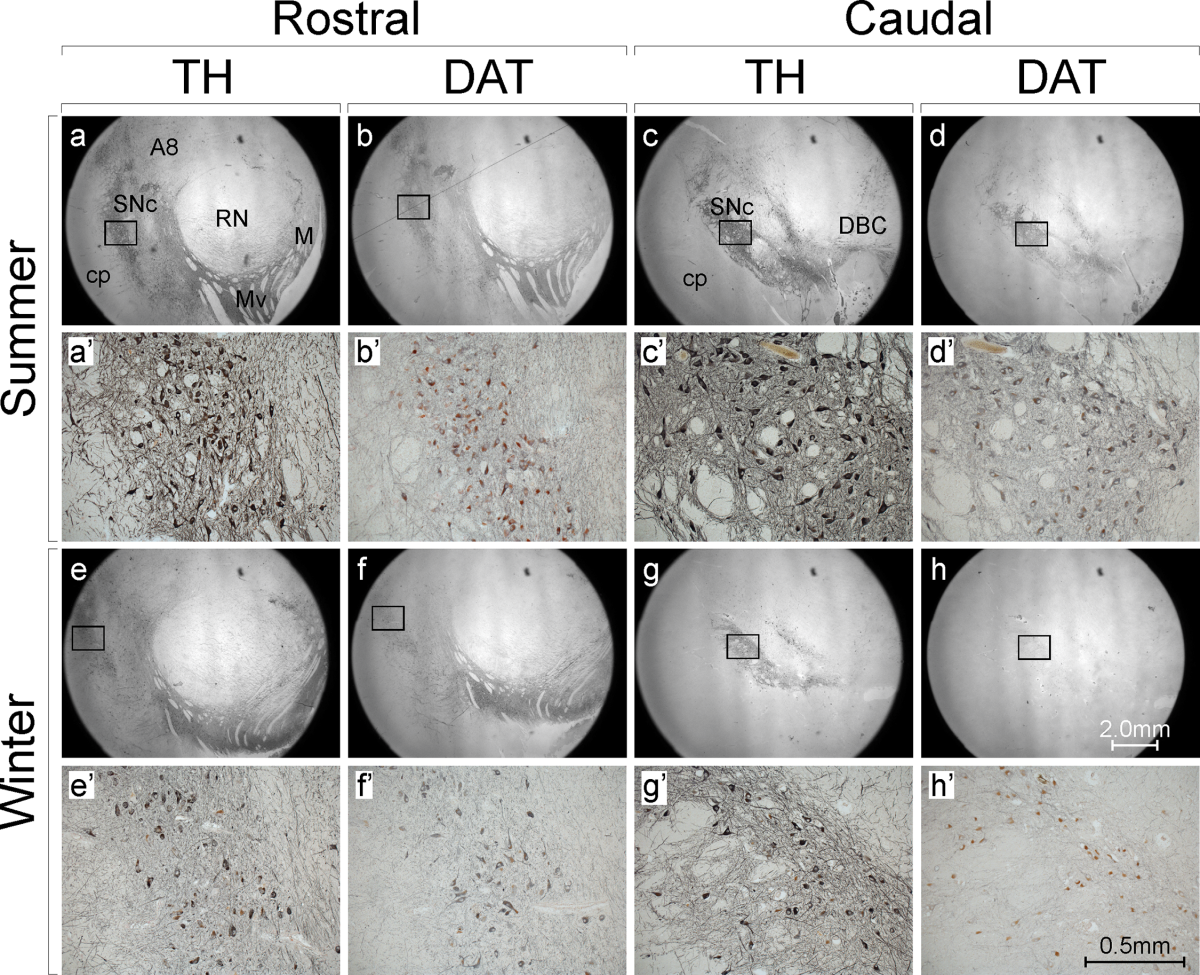
Midbrain tyrosine hydroxylase (TH) and dopamine transporter (DAT) immunoreactivity are higher in summer than in winter. Shown are photomicrographs of immunoreacted (black DAB reaction product) and Nissl-counterstained (red) human post-mortem sections at rostral (**a,b,e,f**) and caudal (**c,d,g,h**) levels of the midbrain in subjects who died in summer (**a,b** = SD033/11; **c,d** = SD030/11) and winter (**e,f** = SD002/10; **g,h** = SD034/10). The regions outlined with squares in **a-h** are shown at higher magnification in **a’-h’**. Note the increased density of immunoreactive (black) cell bodies and neural processes in summer compared with winter, more obvious for TH than DAT. A8 = dopaminergic group A8; cp = cerebral peduncle; DBC = decussation of the brachium conjunctivum; M = medial dopaminergic group; Mv = medioventral dopaminergic group; RN = red nucleus; SNc = substantia nigra pars compacta

Midbrain TH and DAT immunoreactivity was higher in summer than in winter. This was evident at both low and high magnification (e.g. compare Fig 1A–1D with 1E and 1F, & 1A’–1D’ with 1E’–1H’), and was due to more immunoreactivity within immunopositive neurons and processes, as well as more immunopositive neurons and processes in summer (e.g. compare Fig 1A’–1D’ with 1E’–1H’).

### Quantitative TH+, DAT+ and glial cell density comparisons

#### t-tests

**Fig 2:**
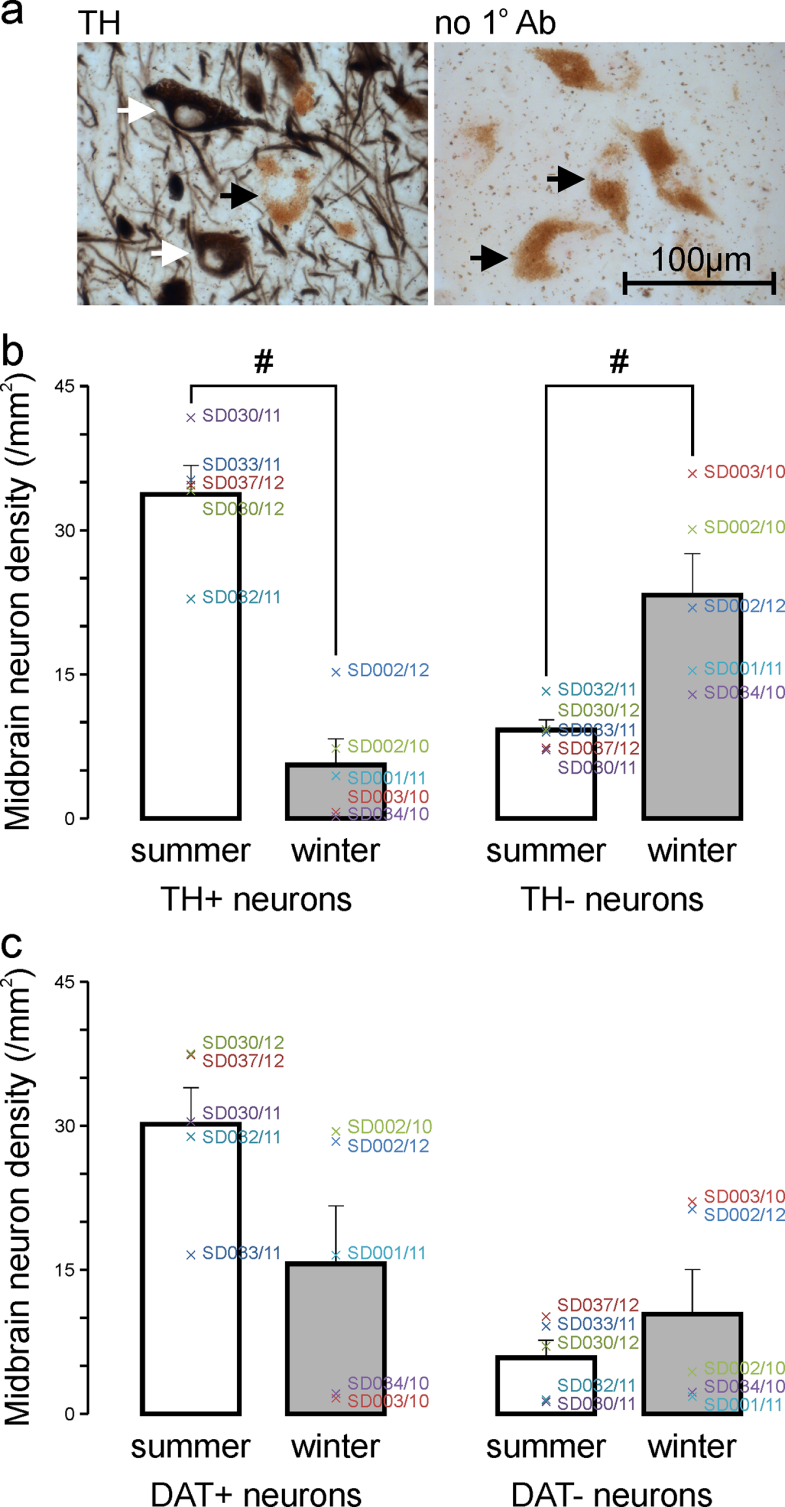
The density of tyrosine hydroxylase immunoreactive (TH+) midbrain neurons is higher in summer than in winter. **a** Cell-type classifications were made during cell density measurements. Only large (neuronal) cells with a visible nucleus were counted. TH+ neurons contained black TH immunohistochemistry reaction product (e.g. white arrows), whereas TH- neurons (e.g. black arrows) had no black reaction product but contained brown clusters of neuromelanin granules around the nucleus and/or Nissl counterstain (red staining). The photomicrograph on the left is of a section processed with the primary TH antibody, the photomicrograph on the right is of a negative control section processed without the primary TH antibody (no 1° Ab). **b** Mean ± SE density of midbrain TH+ (left) and TH- (right) neurons in the summer (white bar) and winter (gray bar) groups (n = 5 in each case). Individual subject means are indicated by colored crosses. TH+ neuron density is significantly higher in summer compared with winter (#, p<0.017, unpaired two-tailed t-test with correction for multiple comparisons), and TH- neuron density is significantly lower in summer compared with winter (#, p<0.017, unpaired two-tailed t-test with correction for multiple comparisons). **c** Mean ± SE density of midbrain DAT+ (left) and DAT- (right) neurons in the summer (white bar) and winter (gray bar) groups (n = 5 in each case). Individual subject means are indicated by colored crosses. Although seasonal differences in both DAT+ and DAT- neurons show the same trends as TH+ and TH- neurons, the differences are not statistically significant (DAT+, p>0.017; DAT-, p>0.017, unpaired two-tailed t-tests with correction for multiple comparisons)

To quantify these differences we randomly and systematically sampled all of the available midbrain DA nuclei in each subject and scored neurons as TH+, TH-, DAT+ or DAT- as detailed in the Materials & Methods. Average cell-type densities for each subject, along with their means and standard errors (SE) for each group (summer and winter), are presented in Tables 2 & 3 and displayed graphically in Fig 2B & 2C. Note, densities are per unit area rather than volume because the thin (8 μm) sections were essentially 2-dimensional relative to the large volume (e.g. Fig 2A) of the counting units (midbrain neurons).

In summer there was, on average, a ~6-fold higher density of TH+ neurons and a ~2.5-fold lower density of TH- neurons compared with winter (Table 2 & Fig 2B). The density of total neurons (TH+ and TH- combined) was not significantly different in summer compared with winter (Table 2). Although there was a ~2-fold higher density of DAT+ neurons and a ~2-fold lower density of DAT- neurons in summer compared with winter, these differences did not reach statistical significance (Table 3 & Fig 2C). Likewise, the density of total neurons (DAT+ and DAT- combined), which was carried out on a different series of sections, was not significantly different in summer compared with winter (Table 3). The density of presumed glial cells (see Materials & Methods) was also not different in summer compared with winter (Table 3).

#### Clustered median regression analyses

Assuming similar age, gender, side of the brain and PMI, the median density of TH+ neurons was 4.36 (95% confidence interval 2.79, 5.92) units higher in summer than winter (p < 0.001); the median density of TH- neurons was 1.65 (95% confidence interval 0.63, 2.68) *lower* in summer than winter (p = 0.002); and the median density of total neurons (TH+ and TH- combined) was 3.04 (95% confidence interval 1.13, 5.00) units higher in summer than winter (p = 0.002).

Assuming similar age, gender, side of the brain and PMI, the median density of DAT+ neurons was 2.61 (95% confidence interval 0.55, 4.68) units higher in summer than winter (p = 0.014); and the median density of total neurons was 2.2 (95% confidence interval -0.86, 5.27) units higher in summer than winter (p = 0.156). The model did not converge when analyzing median density of DAT- neurons.

Assuming similar age, gender, side of the brain and PMI, the median density of glia was 1.11 (95% confidence interval -0.66, 2.88) units higher in summer than winter (p = 0.212).

## TUNEL comparison

**Fig 3:**
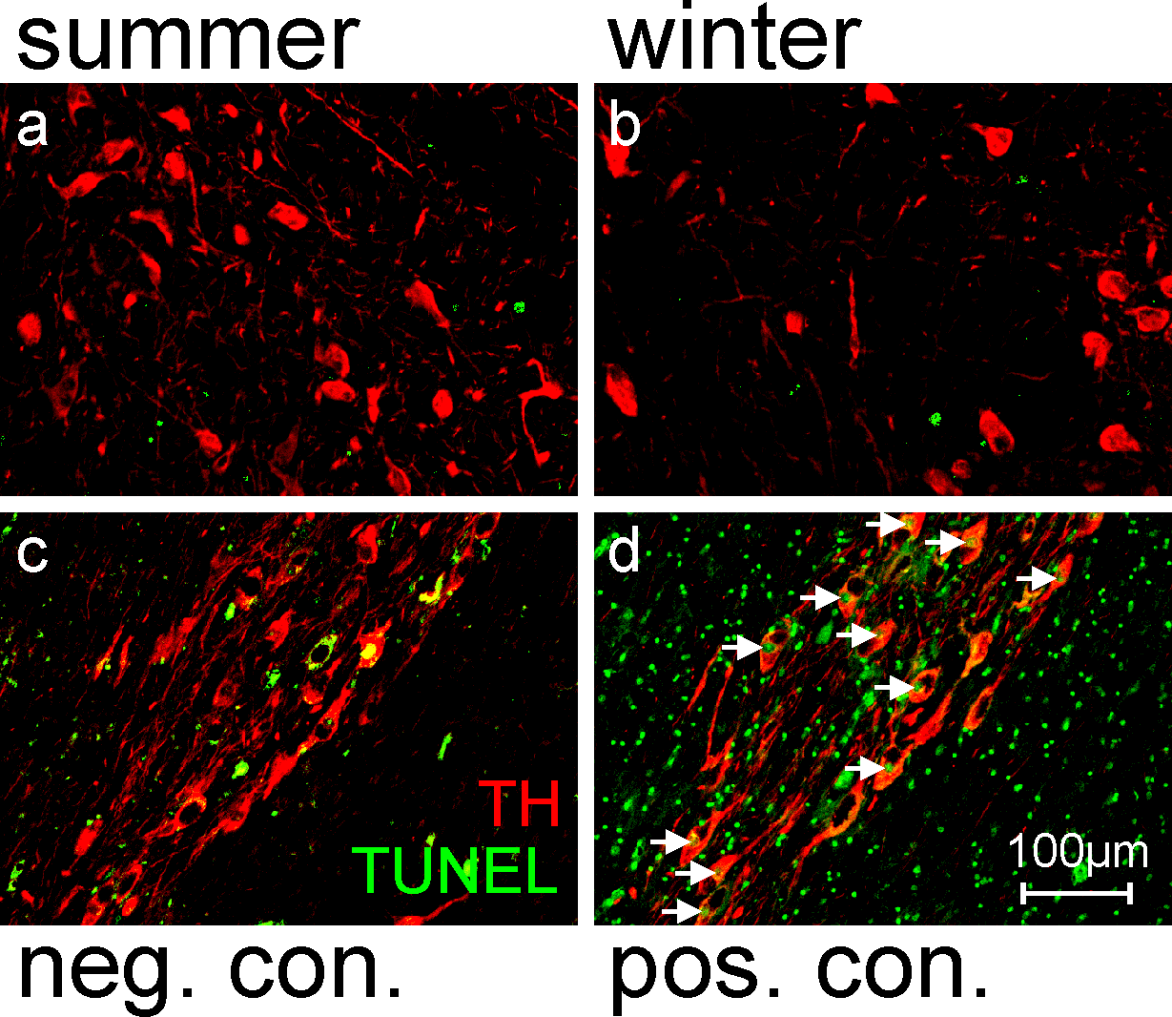
TUNEL staining in midbrain DA nuclei is the same in summer and winter, suggesting apoptosis is not the cause of seasonal differences in TH+ and TH- neurons. **a & b** Photomicrographs of sections through approximately the same area of midbrain in a summer (a) and winter (b) subject. These sections have been immunoreacted for TH (red) and stained for TUNEL (green). Note the increased density of TH+ neurons in summer but no difference in the amount of TUNEL staining between summer and winter. **c & d** Photomicrographs of TUNEL negative (c) and TUNEL positive (d) control sections. Both have been immunoreacted for TH (red), but the TUNEL negative control section was processed without the enzyme component of the kit, and the TUNEL positive control section was treated with DNAse prior to the TUNEL reaction. Note the lack of TUNEL+ nuclei in (c) and the large number of TUNEL+ nuclei in (d), including large TUNEL+ & TH+ nuclei (white arrows) and small TUNEL+ nuclei that are presumably glial cells

To investigate potential causes of these seasonal differences in TH+ and TH- neuron density, we examined the cell-death (apoptosis) marker TUNEL. If cell death underlies the reduced number of TH+ neurons in winter, one would expect more TUNEL+ cells in winter than in summer subjects. However, this was not the case. Rather, the number of TUNEL+ cells in the midbrain DA nuclei was the same in summer compared with winter (Fig 3A & 3B).

## Discussion

This study provides evidence of association between environmental stimuli (photoperiod) and the number of midbrain DA neurons in humans. It also suggests DA neurotransmitter switching underlies this association. We show: (1) Higher TH and DAT immunoreactivity in midbrain neurons and their processes in summer compared with winter; (2) A ~6-fold higher density of TH+ midbrain neurons in summer compared with winter; (3) A ~2.5-fold lower density of TH- midbrain neurons in summer compared with winter; (4) No differences in density of total midbrain neurons (TH+ and TH- combined, or DAT+ and DAT- combined) in summer compared with winter; and (5) No differences in TUNEL staining between summer and winter.

We compared cell density rather than cell number because the tissue blocks supplied did not encompass the entire midbrain DA nuclei, and they were from different rostro-caudal levels. Thus there was substantial non-biological variation in the volume of midbrain DA nuclei available for analysis, which would have transferred to estimates of cell number (i.e. density x volume). In these circumstances cell density better reflects biological variation.

The source of the variability in density of TH+ and TH- neurons reported here is most likely one or perhaps several factors that co-vary with the summer and winter seasons (e.g. photoperiod, temperature, diet, artificial light exposure, physical activity). Other factors such as time of death, subject age, gender, post-mortem interval and regional variations within the midbrain DA nuclei are unlikely to contribute because these were not significantly different between the summer and winter groups, and we did our best to control for these by including them as covariates in the multiple regression model.

The higher TH and DAT immunoreactivities of midbrain neurons and their processes in summer compared with winter (Fig 1) imply higher levels of TH and DAT protein in these cells in summer. Indeed the concurrently higher density of TH+ neurons and lower density of TH- neurons in summer (Fig 2B) is consistent with TH protein levels rising above detection threshold in some midbrain neurons in summer. Conversely the concurrently lower density of TH+ neurons and higher density of TH- neurons in winter (Fig 2B) is consistent with TH protein levels falling below detection threshold in some midbrain neurons in winter. This is what we refer to as ‘DA neurotransmitter switching’, and is the most parsimonious explanation for the present findings. Alternatively, the different densities of TH+ and TH- neurons could be due to combinations of neurodegeneration and neurogenesis. Against degeneration are our findings of no difference in density of total neurons [TH+ and TH- combined, or DAT+ and DAT- combined (Tables 2 & 3)] and no difference in TUNEL+ nuclei (a marker of apoptotic cell death) in summer compared with winter (Fig 3). Neurogenesis is unlikely to contribute given the mismatch between its, at best, indolent rate and the magnitude (6-fold) and speed (<6 months) of the differences we observed [7–14].

While there was a large (~6-fold) and significant difference in density of TH+ neurons between summer and winter (Fig 2B), the smaller magnitude (~2-fold) difference in density of DAT+ neurons (Fig 2C) did not reach statistical significance. DAT is co-expressed with TH in DA neurons. The smaller seasonal difference in density of DAT+ compared with TH+ neurons may reflect differences in transcriptional or translational regulation, differences in post-mortem enzyme stability or differences in antibody affinity.

Our data add to evidence that DA synthesis, turnover, and signaling in the human brain varies with the seasons [15–20,22]. However, the sign of this relationship is unclear, with some studies pointing to higher DA in summer ([15,22], present study) and others pointing to higher DA in winter [16,18–20]. In hypothalamus, Carlsson et al. [17] report a more complex relationship with two peaks (January-February and again in August-September) and two nadirs (March-June and October-December). Lower DA in winter may be contributed to by bright light exposure that is a feature of greater usage of indoor artificial light [2]. The intensity of illumination as well as its duration are likely to have been important. However the use of artificial lights to extend day length would have reduced the difference between summer and winter light exposure, reducing the extent of the difference that we observed.

It is necessary to distinguish between higher and lower DA and more or fewer DA neurons, since the implications for circuit function are different. Positron emission tomography (PET) or single photon emission computed tomography (SPECT) studies focusing on the midbrain DA system have shown higher kinetic rate constants (Ki) of striatal ^18^F-DOPA accumulation in winter [19,20] and lower striatal D2/D3 receptor binding availability associated with marginally shorter (~2 hours) sunlight exposure in the sub-tropics [22]. However, the relationship between these differences and the amount of midbrain DA, or more importantly the amount of midbrain DA signaling is unclear. For example, higher rates of ^18^F-DOPA uptake may reflect lower endogenous L-DOPA or DA, and lower striatal D2/D3 receptor binding availability may reflect down-regulation of D2/D3 receptors in response to lower DA. To be fair, the higher TH immunoreactivity we measured in summer may be a compensatory response to lower rates of midbrain DA synthesis. A further clue comes from knowledge that neurotransmitter switching fundamentally involves changes in levels of neurotransmitters (e.g. [31], cited in [32]) and that this is recognized by the postsynaptic neurons that change the levels of their neurotransmitter receptors to match [1]. Although we did not measure neurotransmitter receptors in the present study, we did find seasonally concordant differences in TH and DAT immunoreactivity. DAT is analogous to DA receptors in so far as it is a plasma membrane protein that binds extracellular DA. These data therefore suggest lower DA and DA signaling in winter compared with summer. Also, the strong association between low DA and depression [23], together with the prevalence of depressive symptoms in winter, are ostensibly more consistent with decreased DA signaling and a smaller number of DA neurons in winter.

In conclusion, this study provides the first evidence of an association between environmental stimuli (photoperiod) and the number of midbrain DA neurons in humans, and suggests DA neurotransmitter switching underlies this association. If this is confirmed in future experiments, other environmental cues that drive midbrain DA neurotransmitter switching in rodents (e.g. neuronal activity, sex-pairing, environment enrichment [3–6]) might do so also in humans.
